# Pravastatin alleviates lipopolysaccharide-induced placental TLR4 over-activation and promotes uterine arteriole remodeling without impairing rat fetal development

**DOI:** 10.7555/JBR.32.20180039

**Published:** 2018-06-30

**Authors:** Muyi Yang, Zhenyu Diao, Zhiyin Wang, Guijun Yan, Guangfeng Zhao, Mingming Zheng, Anyi Dai, Yimin Dai, Yali Hu

**Affiliations:** 1. Drum Tower Clinical Medical College, Nanjing Medical University, Nanjing, Jiangsu 210008, China; 2. Department of Obstetrics and Gynecology, Nanjing Drum Tower Hospital, Nanjing University Medical School, Nanjing, Jiangsu 210008, China.

**Keywords:** preeclampsia, arteriole remodeling pravastatin, toll-like receptor 4, fetal development

## Abstract

Preeclampsia is associated with over-activation of the innate immune system in the placenta, in which toll-like receptor 4 (TLR4) plays an essential part. With their potent anti-inflammatory effects, statins have been suggested as potential prevention or treatment of preeclampsia, although evidence remains inadequate. Herewith, we investigated whether pravastatin could ameliorate preeclampsia-like phenotypes in a previously established lipopolysaccharide (LPS)-induced rat preeclampsia model, through targeting the TLR4/NF-κB pathway. The results showed that pravastatin reduced the blood pressure [maximum decline on gestational day (GD) 12, (101.33±2.49) mmHg *vs.* (118.3±1.37) mmHg, *P*<0.05] and urine protein level [maximum decline on GD9, (3,726.23±1,572.86)μg *vs.* (1,991.03±609.37)μg, *P*<0.05], which were elevated following LPS administration. Pravastatin also significantly reduced the rate of fetal growth restriction in LPS-treated rats (34.10% *vs.* 8.99%, *P*<0.05). Further pathological analyses suggested a restoration of normal spiral artery remodeling in preeclampsia rats by pravastatin treatment. These effects of pravastatin were associated with decreased TLR4/NF-κB protein levels in the placenta and IL-6/MCP-1 levels in serum. Additionally, no obvious abnormalities in fetal liver, brain, and kidney were found after administration of pravastatin. These results provide supportive evidence for use of pravastatin in preventing preeclampsia.

## Introduction

Preeclampsia (PE), a multisystem disorder characterized by new onset of hypertension and proteinuria after 20 weeks of gestation, remains a major cause of maternal, fetal and neonatal morbidity and mortality^[1^‒^2]^. It is well known that the activation of the innate immune response in normal pregnancy is magnified in preeclampsia^[3]^. Toll-like receptor (TLR) 4 is considered one of the most robust signals that triggers the activation of innate immune system^[4]^, Over activation of TLR4 signaling pathway inhibited the extra villous trophoblast cells invasion/migration resulting in insufficient remodeling of uterine spiral arteries, which is the pathological hallmark of PE. Our previous study used the lipopolysaccharide (LPS)-induced PE model in rats^[5]^ to describe the molecular signaling involved in PE pathology and showed that NF-κB is a pivotal downstream effector of TLR4 signal^[6]^. Statins are well-known anti-inflammatory molecules and were shown to antagonize the effects of TLR4/NF-κB in multiple cells *in vitro*^[7^‒^10]^. Several case series reported the safety and effectiveness of statins in prevention of pregnancy-associated complications including PE^[11^‒^13]^. Though the lack of randomized data limits the use of statins in human, the data from animal models could provide valuable evidence^[14]^.

In this study, we hypothesized that pravastatin could improve the PE-like phenotypes in LPS-induced rat model^[5]^ through its anti-inflammation function. We also evaluated the possible side effects of pravastatin on development of fetal brain, liver and kidney.

## Materials and methods

### Animals and the experimental protocol

Ten-week-old female Sprague-Dawley (SD) rats, weighing (250±10) *g* each, were housed in temperature and humidity controlled quarters with constant light/dark cycles of 12 hours/12 hours. They were mated with male rats in a 1:1 ratio overnight. The day when vaginal spermatozoa was observed was designated as gestational day (GD)0. Pregnant rats were randomly divided into three groups: the control group (6), LPS group (6), and LPS-pravastatin group (6). On GD5, rats in the control group were injected with 2 mL of saline into the veins of the tails and the LPS or LPS-pravastatin group were injected with 0.5μg/kg LPS (*Escherichia coli *serotype 0111: B4, Sigma-Aldrich, USA) dissolved in 2 mL saline^[5]^. From GD6 to GD19, the LPS-pravastatin group was treated with pravastatin (Cayman, Michigan, USA, 1001034) 1 mg dissolved in drinking water daily. On GD19, pregnant rats were intraperitoneally anesthetized with 10% chloral hydrate (3 mL/kg). Blood samples were obtained from the heart, left in room temperature for 1 hour until the blood clot solidified, then stored in 4 °C overnight and centrifuged at 3,000 *g* for 10 minutes at 4 °C. The serum was collected and stored at ‒80 °C. The fetus number and fetal resorption rate were recorded, and the wet weights of pups and placentas were measured using electronic balance (Mettler Toledo, Shanghai, China), with actual scale interval 0.01 mg. All procedures and protocols were approved by the Committee on the Ethics of Animal Experiments of Drum Tower Hospital and all animal researches were consistent with the guidelines of Experimental Animals Management Committee (Jiangsu Province, China).

### Measurement of blood pressure, urinary protein level and fetal growth restriction

As previously described^[5]^, the systolic blood pressure (SBP) of the rats was monitored every 3 days (8:00 a.m. to 10:00 a.m.) by tail-cuff plethysmography (BP-98A; Softron, Tokyo, Japan). Briefly, each rat was warmed to 38 °C and SBP was assessed continuously 15 times, in which 3 continuous values of variation of less than 6 mmHg were averaged to define maternal SBP. The rats were trained to be quiet for one week before their mating when blood pressure was measured. The rat urine was collected every 3 days (20:00 p.m. to 10:00 a.m.) with dams housed individually in metabolic cages without food. Urinary protein level was measured by the pyrogallol red method^[13]^ together in one batch after all the samples were collected. Fetal growth restriction (FGR) was defined as fetal weight falling below the 10^th^ percentile in the control group as previously mentioned^[1]^.

### Immunostaining

The fresh rat placenta samples were fixed in 10% formaldehyde, dehydrated in graded alcohol and embedded in paraffin. Placentas with their associated mesometrial triangle were fixed by paraffin, and parallel sections were cut step-serially from each implantation site, parallel to the mesometrial-fetal axis, as described previously^[15^‒^17]^. Myometrial triangle in placenta implantation site was selected sections including a central maternal arterial channel. Cytokeratin (CK) (1:500 dilutions, Rb 15539-1-AP, ProteinTech, Chicago, USA) was used to stain trophoblast invasion, while α-SMA (1:800 dilutions, Rb ab5694, Abcam, Cambridge, MA, USA) was the marker of vascular smooth muscle^[5]^. The degree of trophoblast invasion and spiral artery (SA) remodeling were assessed using Image J analysis system as described earlier^[16]^, briefly, the lumen of each SA cross-section in the whole MT was manually delineated and stretches of trophoblast and vascular smooth muscle were traced separately over the lumen contour tracing, and the percentages of CK staining and α-SMA staining of the corresponding spiral artery contour were calculated. TLR4 and p65 expressions in rat placenta were determined using corresponding antibodies (TLR4, BS3489, 1:100 dilutions, Bioworld Technology, Nanjing, China; p65, 1:200 dilutions, Bioworld Technology, Nanjing, China) to describe the placenta inflammation. Image J analysis system was used to calculate the percentage of positive cells in placenta, 6 placentas per group, and 2 slides per placenta were involved.

### Western blot analysis

Proteins were separated by SDS-PAGE^[5]^. The protein concentrations were measured by Bradford assay (Bio-Rad, Hercules, CA, USA). Immunoblotting was performed with primary antibodies raised against NF-κB p65 (1:1,000 dilutions, BS1253; Bioworld, St Louis Park, MN, USA), TLR4 (1:1,000 dilutions, BS3489; Bioworld, St Louis Park, MN, USA), and β-actin (1:5,000 dilutions, AP0060; Bioworld, St Louis Park, MN, USA), followed by incubation with a goat anti-rabbit horseradish peroxidase (HRP)-conjugated secondary antibody (1:10,000, Bioworld, St Louis Park, MN, USA). Detection was conducted using enhanced chemiluminescence kit (Amersham Biosciences Corp, Piscataway, NJ, USA), and densitometric analysis of each band was undertaken with Quantity-one (Bio-Rad, Hercules, CA, USA) software.

### Cell culture and CTB invasion assay

The HTR-8/SVneo cells were kindly provided by Dr. Charles Graham from Queen’s University, Canada. The cells were maintained in standard culture conditions at 37 °C in a humidified 5% CO_2_ incubator. The culture medium (HyClone, South Logan, USA) was RPMI 1640, supplemented with 10% heat-inactivated fetal bovine serum (FBS), 100 U/mL penicillin and 100μg/mL streptomycin^[5]^. HTR-8/SVneo cells were adjusted to 5×10^5^ cells/well in 6-well plates. Cultured in 1% FBS for 12 hours, the cells were treated with 100 ng/mL of LPS (Sigma-Aldrich, St. Louis, USA) to mimic the inflammation induced PE placenta; additionally, 20μg/mL pravastatin^[18]^ (Sigma-Aldrich, St. Louis, USA) was added as a treatment group. LPS and pravastatin were dissolved in the same RPMI 1640 with 10% FBS.

After 24 hours treatment, the cells were plated in transwell inserts on Matrigel (BD, New Jersey, USA)-coated polycarbonate filters (pore size=8.0μm, Millipore). After 36-hour-incubation, the uninvaded cells were removed with cotton swabs, and the lower surfaces of the filters fixed in 4 g/100 mL paraformaldehyde and stained with 0.2% crystal violet. The data were presented as the number of invaded cells per field viewed using Leica DMR microscope.

### Enzyme-linked immunosorbent assay

Cytokines IL-6 and MCP-1 in rat serum were quantitatively detected by enzyme-linked immunosorbent assay (ELISA) kits (SenBeiJia Biological Technology, Nanjing, China) rat IL-6 (detection range, 8 ng/L‒150 ng/L); rat MCP-1 (detection range, 30 ng/L‒700 ng/L) was directed according to the manufacturers’ instructions.

### Serum total cholesterol measurement

Frozen serum melted on ice, and total cholesterol levels were determined using colorimetric ELISA kits (Kyowa Kirin, Tokyo, Japan) according to manufacturer’s instructions and an automated spectrophotometer (Beckman Coulter, California, America). All samples were measured in one batch.

### Quantitative real-time PCR

Total RNA was extracted using TRIzol reagent (Invitrogen, California, USA). cDNA was synthesized from 1μg of purified RNA using a Prime Script RT Master Mix kit (Takara, Shiga-ken, Japan) according to the manufacturer's instructions. Quantitative real-time PCR (qRT-PCR) was performed and the specific primers (5'-3') used for PCR analysis were listed as: Rat IL-6 F: GCCCTTCAGGAACAGCTATG, R:CAGAATTGCCATTGCACAAC; Rat MCP-1 F: TTCACAGTTGCTGCCTGTAG, R:TCTGATCTCACTTGGTTCTGG; Rat GAPDH F:ATGGGAAGCTGGTCATCAAC, R: GGATGCAGGGATG-ATGTTCT. Data were analyzed by the 2^−ΔΔCT^ method, and GAPDH was used for normalization^[5]^.

### Statistical analysis

The incidence of FGR and resorbed fetus were analyzed by chi-square test, Fisher's exact test and factorial. Other data were presented as mean±SD. ANOVA and Student-Newman-Keuls tests were performed for the comparison of multiple groups. A two-tailed *P*-value of less than 0.05 was considered significant. Data analysis was accessed by Microsoft Excel 2016 and GraphPad Prism 7 software.

## Results

### Pravastatin ameliorates hypertension, proteinuria and FGR in a LPS-induced PE-like rat model

**Fig.1:**
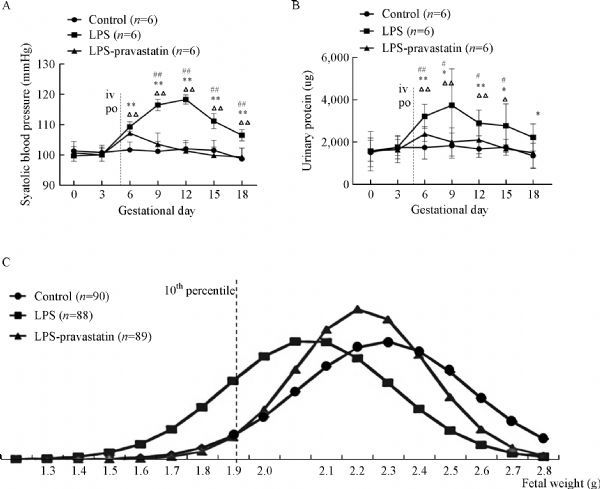
Systolic blood pressure, urinary protein and fetal weight of each group on different gestational days. Pregnant rats were randomly treated with normal saline, LPS, or LPS plus pravastatin as detailed in Methods. Systolic blood pressure (SBP) (A) and urinary protein (B) of each group on different gestational days (GD). Data are presented as mean ± SD. ^Δ^P < 0.05, ^ΔΔ^P < 0.01 vs. GD0; *P < 0.05, **P < 0.01 vs. the control group on the corresponding GD; #P < 0.05, ##P < 0.01 vs. the LPS-pravastatin group on the corresponding GD. The NORMDIST function was used to normalized the data. The normal distribution curve was used to describe the fetal weight (C), and the curve moved to the left after administration of LPS compared to the control group, and then shifted to the right after administration of pravastatin. The cutoff value curve is defined as the fetal weight falling below the 10th percentile in the control group.

To activate TLR4 pathway, we injected low-dose LPS into pregnant rats in GD 5 through tail veins. Compared with control group [(101.67±2.36) mmHg], a significant elevation in systolic blood pressure started from GD6 in LPS group [(109.33±1.49) mmHg, *P<*0.05], and this elevation continued until GD18 (***Fig. 1A***).The concentrations of urinary protein increased from GD6 to GD18 in LPS group, most significantly on GD9 [(3,726.23±1,572.86)μg] compared to (1,820.12±541.84)μg in control group, *P*<0.05 (***Fig. 1B***). However, in LPS-pravastatin group, the SBP dropped to (103.50±3.40) mmHg from GD9 and maintained the similar level to control group, although at GD6 the SBP rose to (107.17±1.77) mmHg (*P*<0.05) (***Fig. 1A***).The urinary protein concentrations had the same tendency as the blood pressure. Pravastatin decreased the elevated level of urine protein induced by LPS (***Fig. 1B***).

The normal distribution curve (***Fig. 1C***) was used to describe the fetal weight and there was a larger area on the left of the 10% cutoff line in the LPS group than those of the other two groups suggesting decreased average weight of the fetuses in LPS group and restored by pravastatin. Pregnant outcomes indicated that the incidence of FGR in pravastatin group (8.99%) was similar to that in negative control group (8.89%) and significantly lower than this in LPS group (34.10%). The fetal resorption rate was zeroin both LPS-pravastatin group (0/89) and control (0/90) group. However, in the LPS group, the rate of fetal resorption showed an increasing tendency 3/91, although the *P *value was not significant.

### Pravastatin facilitates trophoblast cell invasion

**Fig.2:**
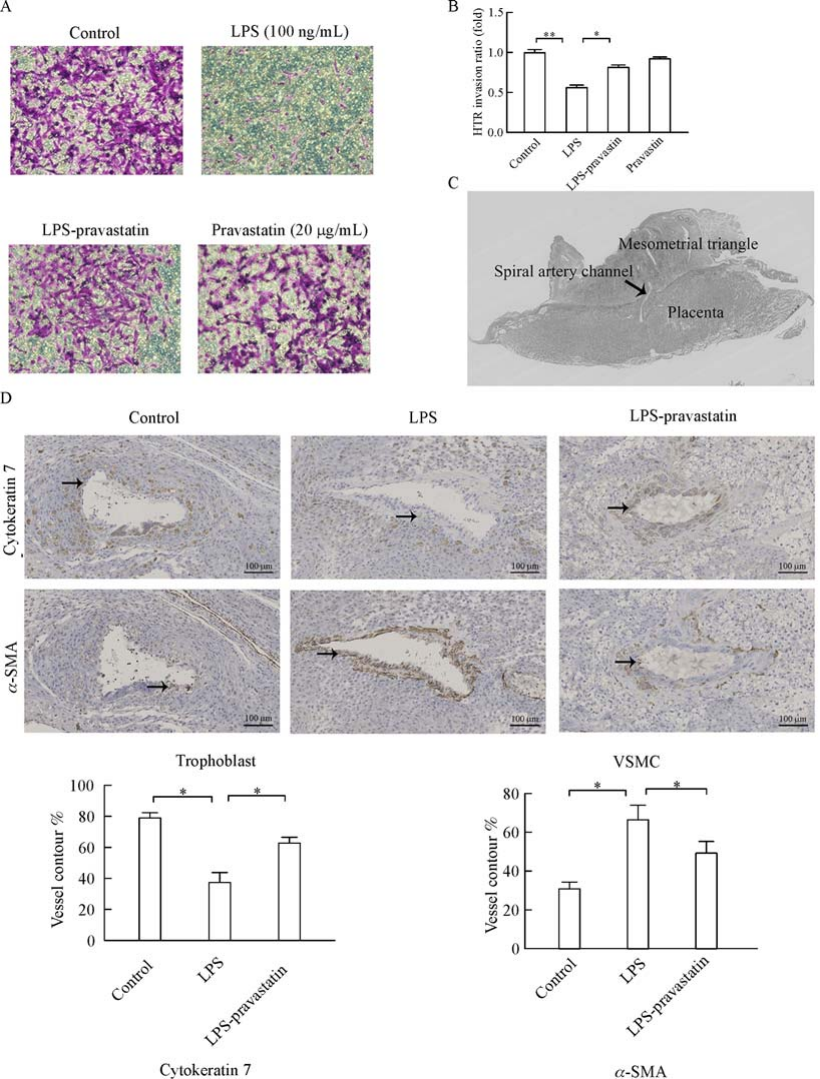
Pravastatin alleviates LPS-induced suppression of trophoblast cell invasion. Pravastatin obviously improved trophoblast cell invasion inhibited by LPS in vitro, and there were significant differences among groups (P < 0.01) (A–B). Data were presented as mean ± SD. The representative placenta immune-stained site with a spiral artery (SA) channel and the associated mesometrial triangle (MT) were taken (C). Representative trophoblast cell invasion (cytokeratin, solid arrows) and VSMC (α-SMA, solid arrows) in the SA of MT. The percentages of trophoblast cell and VSMC of total SA contour length were recorded. Six placentas per group, and 2 slides per placenta were examined to calculate the percentage of positive cells in the placenta. There was significant difference among three groups (P < 0.01), *P < 0.05, **P < 0.01 between two groups. Magnification 200 ×. Bar 100 μm (D). VSMC: vascular smooth muscle cell.

Cell invasion experiment *in vitro* using HTR-8/SVneo cells showed that LPS significantly inhibited trophoblast invasion (0.57 fold compared to control group, *P*<0.05), while pravastatin restored the inhibition of trophoblast invasion induced by LPS (0.82 fold, *P*<0.05) (***Fig. 2A‒B***).

Next, the trophoblast invasion and spiral artery (SA) remodeling in rat were assessed in the myometrial triangle. The percentages of trophoblast and vascular smooth muscle cells (VSMC) of total SA contour length were determined. There were more cytokeratin positive trophoblast cells (63.6%) and less α-SMA labeled smooth muscle cells (49.3%) in the LPS-pravastatin group than those in LPS group (cytokeratin positive trophoblast, 37.3%; α-SMA labeled smooth muscle cells, 66.7%), while the levels of CK7 and α-SMA were 79.0% and 31.3% respectively in control group (***Fig. 2D***). Pravastatin improved the undermined trophoblast invasion and SA remodeling in PE-like rat model.

### Pravastatin suppresses LPS-induced over-activation of the TLR4/NF-κB pathway

**Fig.3:**
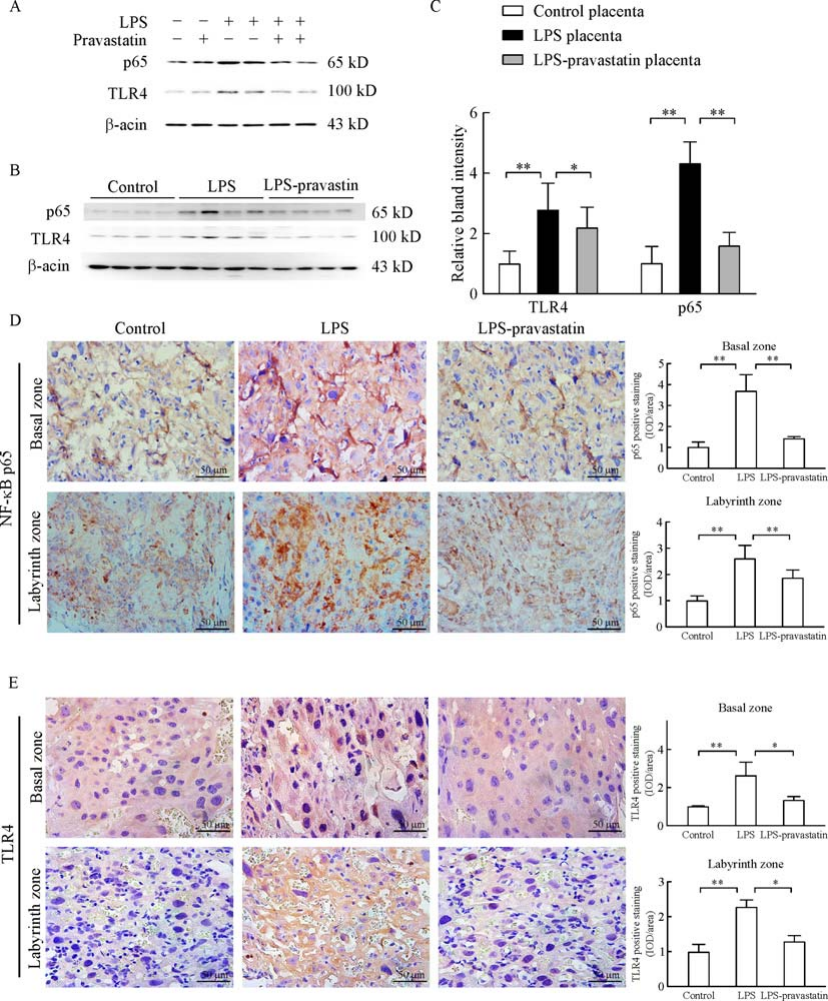
Pravastatin alleviates LPS-induced activation of TLR4 pathway *in vitro* and *vivo.* Western blotting for TLR4 and p65 in control, LPS and LPS-pravastatin and pravastatin treated HTR-8/SV neo cells (A) as well as the rats’ placentas (B–C), 2 placentas per rat, 6 rats per group. Data are presented as mean ± SD. There were significant differences among groups (P<0.01), *P<0.05, **P<0.01 between two groups. Immunohistochemistry for TLR4 and p65 in the placenta of different groups. Six placentas per group, and 2 slides per placenta were examined to calculate the percentage of positive cells in the placenta, and there was significant difference among three groups (P < 0.01), *P < 0.05, **P < 0.01 between the two groups. Magnification 400 ×. Bar 50 μm.

**Fig.4:**
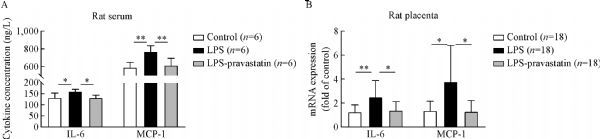
Expression of IL-6 and MCP-1 in the serum and placenta in different groups. Data were presented as mean ± SD. Concentrations of IL-6 and MCP-1 in the serum from different groups were measured by ELISA (A), and there were significant differences among three groups (IL-6, P<0.05; MCP-1, P<0.01), 6 rats per group. *P<0.05, **P<0.01. The mRNA levels of IL-6 and MCP-1 in the placenta from different groups (B) were measured by qRT-PCR and normalized to that of GAPDH, 3 placentas per rat, 6 rats per group. There were significant differences among three groups (P<0.01), *P<0.05, **P<0.01.

HTR-8/SV neo cell line was used to observe pravastatin’s function in trophoblast *in vitro*, and the result of Western blot demonstrated that the high level of TLR4 and NF-κB-p65 protein expression induced by LPS decreased after pravastatin treatment (***Fig. 3A***). The study *in vivo* further proved that pravastatin treatment alleviated the upregulation of TLR4 and NF-κB-p65 in both placental basal zone and labyrinth zone after LPS administration (***Fig. 3B*** ***‒*** ***E***). To verify the role of pravastatin in inhibiting LPS-induced inflammation, relative cytokine levels in rat serum were assessed. ELISA assay indicated that IL-6 and MCP-1 serum levels in LPS group were significantly increased compared to those in control group (*P*<0.05), meanwhile these cytokine levels decreased after pravastatin treatment (*P*<0.05, ***Fig. 4A***). The mRNA levels of IL-6 and MCP-1 in placenta were measured by qRT-PCR and the results consisted with the serum protein results (***Fig. 4B***). The above results indicated that there may be a connection between placenta and systemic inflammatory over-activation.

### Pravastatin does not compromise fetal development or significantly reduce serum total cholesterol level

**Fig.5:**
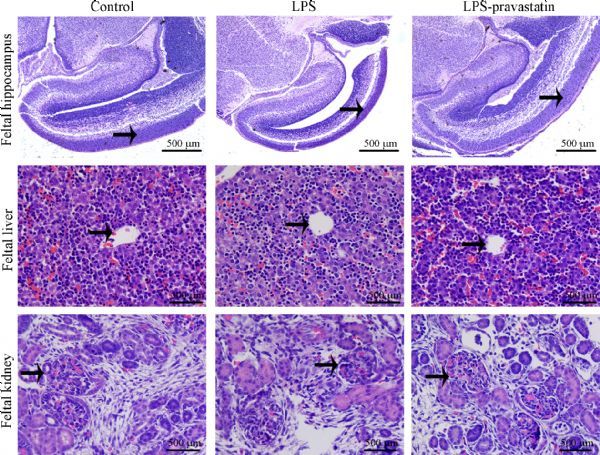
Pravastatin does not significantly compromise the development of main fetal organs. The representative hematoxylin-eosin staining of fetal structures. The hippocampal pyramidal cells (solid arrows), liver lobule (solid arrows) and glomerulus sections (solid arrows) of the fetal rats were chosen to evaluate the structures of fetal main organs. Magnification 200 ×. Bar 100 μm.

As an anti-cholesterol drug, the effects of pravastatin on maternal weights and fetal development were investigated. Firstly, the maternal weight was recorded and had no significant difference on GD5, the interference day, and GD19 (*P*>0.05) (***Table 1***). Secondly, the maternal serum total cholesterol levels had no difference at GD19 among negative control [(1.07±0.31) mmol/L], LPS [(1.07±0.47) mmol/L] and LPS-pravastatin groups [(1.08±0.47) mmol/L)], *P*>0.05. The average fetal weight was heavier in LPS-pravastatin group [(2.24×10^3^±187.08) mg] than that in LPS group [(2.08×10^3^±241.54) mg, *P*<0.05], which was similar to this in control group [(2.30×10^3^±234.07) mg] (***Table 1***). Additionally, we collected cholesterol metabolism-associated or cholesterol abundant fetal organs such as liver, brain and kidney, and examined their weights and structure respectively. The fetal organ size was smaller in the LPS group, and the size improved after pravastatin treatment. The average weight of fetal brain was heavier in LPS-pravastatin group [(117.87±12.37) mg] than that in LPS group [(110.75±9.74) mg, *P*<0.05], which was similar to this in control group [(118.53±11.18) mg]. The hippocampal pyramidal cells morphology was observed to describe the brain development and there was nothing obviously changed. Additionally, pravastatin also improved the average weight of fetal liver [(149.12±26.99) mg] compared to (127.37±22.36) mg in LPS group (*P*<0.05), and it was (153.22±29.95) mg in control group. HE dying was performed without detecting significant change of the number and structure of liver lobule. The average weight of fetal kidney was heavier in LPS-pravastatin group [(5.45±1.29) mg] than that in LPS group [(4.09±1.24) mg, *P*<0.05], which was similar to this in control group [(5.50±1.67) mg] (***Table 1***). Paraffin section focused on the glomerulus and showed no obvious difference among the three groups (***Fig. 5***).

**Tab.1:** The outcomes of rats in different pregnant groups.

						*P*-value	
Variables	Control	LPS	LPS-pravastatin		LPS *vs*.	Control *vs*.	
				Control	LPS-	LPS-	among three
				*vs*. LPS	pravastatin	pravastatin	groups
	6	6	6				
Maternal Weight (GD5) (*g*)	273.67±4.35	263.17±27.73	268.33±24.98	n.s.	n.s.	n.s.	n.s.
Maternal Weight(GD18) (*g*)	338.17±11.95	329.17±32.12	334.17±18.20	n.s.	n.s.	n.s.	n.s.
Serum TC (mmol/L)	1.07±0.31	1.07±0.47	1.08±0.47	n.s.	n.s.	n.s.	n.s.
	90	88	89				
FGR (%)	8.89 (8/90)	34.10 (30/88)	8.99 (8/89)	**	**	n.s.	**
Resorbed Fetus (%)	0 (0/90)	3.30 (3/91)	0 (0/89)	n.s.	n.s.	n.s.	n.s.
Fetal Weight (mg)	2.30*10^3^±234.07	2.08*10^3^±241.54	2.24*10^3^±187.08	**	**	*	**
Fetal Brain Weight (mg)	118.53±11.18	110.75±9.74	117.87±12.37	**	**	n.s.	**
Fetal Liver Weight (mg)	153.22±29.95	127.37±22.36	149.12±26.99	**	**	n.s.	**
Fetal Kidney Weight (mg)	5.50±1.67	4.09±1.24	5.45±1.29	**	**	n.s.	**

FGR: fetal growth restriction; TC: total cholesterol. Data were presented as mean±SD, n.s *P*>0.05, *P<0.05, **P<0.01.The three resorbed fetus in LPS group were only included when calculating the percentage of resorbed fetus, and were excluded when calculating the percentage of FGR and fetal weight.

## Discussion

Over activation of TLR4 signaling pathway has been well described in PE placenta^[19^‒^21]^. We employed the LPS-induced PE rat model because we thought this model has the advantage of highlighting the role of a non-specific inflammation in PE and could be reasonably the treatment target of statins^[5]^. The administration of pravastatin dated from GD5, the day after implantation^[22]^, which means that the treatment covered the whole window of placenta and embryo development. Our results demonstrated that pravastatin decreased the hypertension, urine protein level, and the incidence of FGR of PE-like rat model by ameliorating the exacerbated TLR4/NF-κB activation in placenta from LPS rats’ model. The changes of serum proinflammorty cytokines including IL-6 and MCP-1, as well as improved spiral artery remodeling were consistent with a protective role of pravastatin in PE rats. In sum, pravastatin can target TLR4 to exert its anti-inflammation function in LPS induced PE-like rat model.

Our results are in line with previously published study of statins in different PE animal models. Dong X *et al. *found that simvastatin alleviated l-NAME-induce PE rat model by restoring pro- and anti-angiogenic balance^[23]^. This suggests that multiple mechanisms may be involved in statin-mediated protection against PE. Although having been suggested, yet to our knowledge, the current work for the first time described the anti-inflammatory role of pravastatin in inflammation-related PE model, providing evidence of use of statin to prevent PE.

Currently statins are considered as category X drugs for their inhibition of cholesterol synthesis during embryonic development^[24]^. In human placenta, cellular functions depend primarily on isoprenylation of proteins, a process that is inhibited by statins. Early *in vitro* studies using human placental explants demonstrated detrimental effect of statins on placental growth^[25]^. However, recent *in vivo* and *in vitro* studies did not identify any statin-related impairment of placental function. In these studies, pravastatin was preferred over simvastatin, for its hydrophilic nature, which allows only minimal transfer across placenta.

In our study, we chose the dosage (1 mg/day) of pravastatin according to the reports on mice^[26^‒^28]^ and rats^[29]^ and evaluated the side effects on the fetus of rat model from GD5. Brain is enriched in cholesterol which is indispensable for neurodevelopment. Previous studies suspected that pravastatin prevented fetal cortical brain developmental deficiency induced by complement component C5α in preterm birth model^[30]^. In present study we chose hippocampal to analyze the brain development specifically. Hippocampalis, part of the limbic system, plays a crucial role in cognitive functions such as learning, memory storage, and spatial orientation, and is a metabolic active structure^[31]^. Additionally, pyramidal cells were chosen to evaluate hippocampus development as pyramidal cells engaged in processing spatial and locational information^[32]^. The brain weight was improved after pravastatin treatment compared to the LPS group, then the histology examination tests showed that there was no obvious difference among three groups on the structure of pyramidal cells, indicating pravastatin not damaging the brain development. The liver was predominantly responsible for the cholesterol synthesis in statins treated animals^[33]^. HE staining revealed the structure of liver lobule was normal. We also checked glomerulus and did not found obvious changes in the structure.

In conclusion, pravastatin taken from GD5 could improve the LPS induced PE-like phenotype, including hypertension, proteinuria and shallow trophoblast invasion, improving pregnancy outcomes without obvious pathological changes in vital organs of fetus. We speculate that immune modulation of PE by pravastatin may but not only rely on TLR4 signaling pathway. Further after-birth growth assessment is required. Although the use of pravastatin appears to be beneficial in our study, its benefits need to be investigated in a large, adequately powered randomized-controlled trial before it becomes part of routine clinical practice^[14]^.
